# Quantifying Ecological Literacy in an Adult Western Community: The Development and Application of a New Assessment Tool and Community Standard

**DOI:** 10.1371/journal.pone.0150648

**Published:** 2016-03-03

**Authors:** Sheryn D. Pitman, Christopher B. Daniels

**Affiliations:** 1 School of Natural and Built Environments, University of South Australia, Adelaide, South Australia, Australia; 2 Barbara Hardy Institute, University of South Australia, Adelaide, South Australia, Australia; University of Westminster, UNITED KINGDOM

## Abstract

Knowledge and understanding about how the Earth functions and supports life create the foundation for ecological literacy. Industrialisation, urbanisation and population growth have resulted in changed relationships between many human communities and the natural world. A potential consequence is a compromised capability to make well-informed decisions about how to live sustainably. To gain a measure of ecological literacy within the South Australian community, we collaborated with senior scientists and educators to develop and apply an instrument with the capacity to determine indicative levels of ecological knowledge and understanding. A formal, variable credit, multiple-choice assessment instrument was distributed online to groups and individuals within diverse community sectors and industries. Quantitative analyses of scores indicated that levels of ecological knowledge and understanding within a self-selected sample of over one thousand individuals ranged from very low to extremely high, with the majority of respondents achieving moderate to high scores. This instrument has a demonstrated capacity to determine indicative levels of ecological literacy within and between individuals and groups. It is able to capture mastery of ecological knowledge and understanding achieved through both formal and informal pathways. Using the results, we have been able to establish a range of standards and an aspirational target score for the South Australian community. The value of this work is in its potential to deliver insights into relationships between humans and the rest of the natural world, and into characteristics of eco-literate individuals and communities, that might not otherwise emerge.

## Introduction

This paper explores the concept of ecological literacy and describes the development, application and initial results of an instrument capable of providing an indicative assessment of the level of knowledge and understanding of ecological systems and processes within a self-selecting sample of the adult community of South Australia.

### Ecological literacy: what does it mean?

While the extent and consequences of impact by industrialised societies on nature have been debated for many decades [1], interest in the concept of ecological literacy has only emerged relatively recently. The term ‘ecological literacy’ appears to have been first used publicly in 1986 by Paul Risser in an address to the Ecological Society of America [2]. As a response to a perceived lack of scientific literacy amongst the American public, Risser urged ecologists to consider the essential elements of basic ecological literacy and then to promote an ecology-based literacy to their students and communities [3]. In 1992 David Orr proposed that achievement of a sustainable human society was inextricably linked to ecological literacy and that the ecological crisis reflected a crisis in education [4]. The concept of ecological literacy, since that time, has continued to evolve within scientific and educational communities. Ecological literacy was the theme of the 93rd Annual Meeting of the Ecological Society of America (ESA) in 2008, from which the question arose: what should every citizen know about ecology? The authors speculated that while the level of ecological literacy within the population of the United States and other countries was not known, “there is widespread concern that it is too low to enable effective social responses to current problems” ([5] p495). While the making of, and approaches to, knowledge are complex discussions in themselves, it is now widely accepted that the field of ecological literacy incorporates a range of disciplines [5–6] and forms an important foundation for the broader concepts of environmental and scientific literacy [7–8].

Ecological literacy has been defined as “the ability to use ecological understanding, thinking and habits of mind for living in, enjoying, and/or studying the environment” ([9] p228) and as focusing on the “key ecological knowledge necessary for informed decision-making, acquired through scientific inquiry and systems thinking” ([3] p3). While there is variation in definitions and in the design of frameworks for ecological literacy, knowledge and understanding about nature and how ecological systems work are widely accepted to form the basis of ecological literacy.

### Ecological literacy and sustainability

Critical to the health and survival of any human society is knowledge and understanding of the natural ecological systems that underpin life. Ecological systems, or ecosystems, are described as “systems of organisms interacting with each other and their environment within some spatial and temporal boundaries” ([10] p627). Ecosystems range from large to very small systems. They include complex patterns of interconnected processes and are governed by the laws of thermodynamics [10].

Until recently ecosystems have been considered to refer to systems unchanged by humans. Urban ecology, however, is now an accepted branch of ecological study and urban ecosystems include cities, suburbs and towns [11–12]. Ecosystems, their processes, functions and components (such as plants, animals, soil, water and air) as well as the collective of processes (geological, evolutionary, biophysical and biochemical) that contribute to the Earth as we know it are all encompassed within the broader concept of ‘nature’ [13]. While the science and theoretical framework of ecology continue to evolve, practical knowledge of ecological processes has been an essential requirement for healthy and successful human societies of the past [8], and remains an essential requirement for current and future human societies.

Despite multiple interpretations [14], the concept of sustainability refers in essence to the ability to sustain, or to maintain a functional state. The sustainability of human communities and settlements is intimately related to the sustainability of the rest of nature, including the integrity of ecosystems and their processes [10]. The links between sustainability and the level of ecological knowledge and understanding within human communities are abundantly clear [4,15–16]. Also considered key to sustainability is an understanding of the changes that have taken place in relationships between humans and the rest of nature [17–18].

Rapid growth in industry, technology, agriculture, urbanisation and population has signalled arrival of the new human-centred and human-dominated era known as the Anthropocene [1,8]. Together with changes in the way human societies are organised and accommodated, it can be argued that an increasingly smaller proportion of people have direct contact with fundamental and vital natural processes. Numerous studies and reports have highlighted the detrimental effects of lifestyles with little contact with nature, deemed by many to be a major crisis of our time [19–20]. Participation in and knowledge of the natural world is considered essential for health and development in diverse ways [21–24]. A feature of the Anthropocene, however, is that intimate contact between people and nature has diminished, and knowledge and understanding of the natural environment, once a fundamental survival mechanism, has become a speciality of the relative few.

During the past two decades considerable thought has been given to how ecological literacy can be defined and what it means to be an ecologically literate person [3,5–6, 9,25]. Both local place-based knowledge [4,6,9,22,26–27] and an understanding of the interconnectedness of local and global ecological systems, including the interface of these systems with human society [5,8–9,28] are considered essential. Knowledge and understanding of “the fundamental interconnectedness between humanity and nature” ([29] p 35) and knowledge of the ecological principles that sustain the web of life [15,28] are recurring themes in discussions and evaluations of ecological literacy. In essence then, an ecologically literate person is aware of the interconnected nature of the Earth and all its parts, has the capacity to understand and respond to the ecological relationships between places and their inhabitants, including human beings, and can make informed decisions about how to live in a sustainable manner.

### Measuring ecological knowledge and understanding

Throughout the world several survey instruments have been developed to measure specific aspects of environmental or ecological knowledge. Most have been designed for use with students in schools or in higher education organisations, rather than for use in the general adult population. A small number of relevant adult studies contain valuable results and recommendations [30–36]. Survey tools have also been developed to capture information about attitudes and beliefs. The New Ecological Paradigm, a redesign of the New Environmental Paradigm first developed in 1978 [37] in Washington (USA) to capture environmental attitudes and beliefs [38], has been used internationally. However, while a small number of survey instruments have been used with adult population samples for a variety of related purposes, there have been no broad scale determinants for the specific assessment of ecological knowledge and understanding in the wider adult community [5]. Within Australia there are no published reports of such studies or assessments among adult populations.

This research was designed to address a number of questions arising from exploration of the scholarship and literature in the field of ecological literacy. We asked three questions: is it possible to capture and portray a picture of ecological knowledge and understanding among adults in South Australia; how can ecological knowledge and understanding be assessed in an efficient and meaningful way; and what can we learn from an assessment of ecological knowledge and understanding within a section of the South Australian community? The next steps included the development of an assessment instrument and an initial quantitative analysis of the results generated by its application.

## Methods

Our objective was to develop a measurement tool capable of assessing and reporting an indicative level of ecological knowledge and understanding within the adult community of South Australia (SA). SA is a large state (983,482 square kilometres) in the centre-south of Australia. It is the fourth largest of Australia's states and territories and has a population of over 1.6 million people drawn predominantly from European origins. It has a small indigenous population (less than 3%) and a small but steadily increasing Asian, Indian and African population. The majority (over 1.3 million) of South Australians reside in and around one city (Adelaide), and the state is rated very highly on most world indices of socio-economic advantage.

### Factors in assessment method and design

Several factors relevant to adult assessment were considered. First, assessing the knowledge of adults in contemporary societies is a challenging task largely because of the diversity of ways in which adults have acquired knowledge during the course of their lives and also because of the complexity of social and cultural environments [39]. An assessment instrument therefore needs to take account of the fact that knowledge in adult communities is derived from multiple formal and informal sources and processes [40–41]. Secondly, the assessment process needed to be inclusive. The objective was to test knowledge and understanding attainable through a diverse combination of life experience, informal and incidental learning, observation and general education, rather than merely through formal science education. It was expected that regardless of social, economic, cultural or political context, an individual who was aware of, observed, studied, lived or worked in close connection with the natural world might demonstrate high ecological literacy. Alternatively an individual who was well educated but not actively engaged or connected with the natural world might demonstrate low ecological literacy. The language used in the questions and answers was intended to be accessible to people with a reasonable command of the English language.

Additional factors that influenced the design and format of our instrument included the need to gather and process information effectively and efficiently, regard for the perceived time constraints of much of the adult community in this country, and acknowledgment of the widespread use of and access to computers and online systems. It was noted that an assessment instrument should not be a speed test as proficient respondents will not necessarily be the fastest; test-taking speed has been shown to have little relationship to the level of understanding of the “content universe” ([39] p239).

Research suggests that one of the most effective and efficient types of test employs fixed-response items such as multiple-choice (MC) which are highly regarded as a valuable and generally applicable test form [42–43]. Multiple-choice tests are attractive because they can sample from many content areas, measure a range of cognitive skills and are reliable [43]. They work because the requirement to discriminate between alternatives necessitates a level of mastery of the subject matter [39]. They also have the advantages of objectivity, simplicity and automatic scoring [42]. Taking into consideration these qualities, together with the parameters of the study, it was determined that judicious development of a multiple-choice instrument, accompanied by a range of relatable socio-demographic and psychographic questions, would constitute an appropriate approach.

Best-answer types of MC items are able to measure the level of understanding of abstract concepts so that it is possible to determine whether or not a concept is mastered. Items appear to be ambiguous to those who have an incomplete understanding of the concept being tested and such extrinsic ambiguity is a desirable characteristic of a test [39]. In the wording of question stems and answer choices, however, ambiguity (intrinsic) should be avoided as it creates confusion [44]. To construct quality MC items it is therefore important to devise clear, plausible and attractive distracters [39].

To facilitate a relatively sensitive analysis of ecological knowledge and understanding, we designed a scoring system that discriminated between correct, partially correct and incorrect answers by according ‘variable credit’ ([39] p240). In the expectation that respondents would have differing levels of knowledge and understanding, options that reflected such variance were incorporated, since these have the potential to generate a more accurate measurement than the more commonly used correct response method, referred to in test measurement literature as ‘number correct’ or NC [43]. It was intended that for people with a thorough understanding of the subject matter or concepts within items, correct answers would appear clear, while for those with a limited understanding, answer options would appear ambiguous.

Each question stem had five answer options. One best or ‘true’ answer in each item represented full knowledge and attracted four points (4). One or two ‘partially correct’ answers represented partial knowledge and attracted two points (2). One or two ‘marginal’ answers represented irrelevance or absence of knowledge and attracted zero points (0). In addition, we also provided one incorrect answer that attracted negative points. This ‘exception’ answer represented incorrect knowledge and attracted negative two points (-2). Employment of the -2 value for the exception answer was intended to further reduce the chance of random responses achieving a high score.

### Review of the subject matter: ecology and ecological principles

While variously defined [45], ecology is essentially the study of interactions of organisms with each another and with the physical environment [10] and entails a holistic understanding of natural processes [46–47]. The science of ecology encompasses investigation into the natural world from the viewpoints of several disciplines and by employing various techniques. It includes the study of populations of single species, the way populations interact within communities, and interactions between communities. It also includes the movement of matter and energy within and between ecosystems, and global patterns within the biosphere [46,48]. Ecology has been described as a science of emergent phenomena [11] in which humans, as living organisms, are not only active players [10] but a dominant component.

Consideration of different approaches to organising ecological principles and subject matter [9–10,35,48–50] identified a set of seven key ecological principles: ecosystem processes (including interconnections and interdependence) and services; biological diversity (including genes and gene flow, species and populations); habitats (including biotic and abiotic components) and food webs; threatening processes (anthropogenic and non-anthropogenic) and extinction; evolution, adaptation, resilience and change; energy, water and climate patterns and systems; and human interaction with environment (interdependence, impacts and conservation). These were validated as appropriate by an erudite scientific reference group (SRG) of senior South Australian-based scientists, educators and research fellows in ecology and related fields. The SRG was selected by the research team because of their acknowledged expertise as senior scientists and educators. Their participation was voluntary and with informed consent, covered by Ethics protocol 022806. From these principles test items were developed.

### Item development

In developing the assessment questions these ecological principles were applied to a selection of local, national and global environments. Relevant and contemporary issues considered by the scientific reference group to be important components of ecological understanding were incorporated. This approach necessitated a wide-ranging and trans-disciplinary research review consisting of scholarly literature, reports, journals, books, articles and personal communications. An initial sixty-item pool comprised more topics and questions than required for the actual assessment. Each item addressed one or more of the identified ecological principles. These were reviewed by the SRG for accuracy, clarity and relevance to ecological principles and the assessment objective.

The modified and refined items were tested on 60 adult volunteer university students including both undergraduate and postgraduate students in a variety of courses within the University of South Australia’s School of Natural and Built Environments. Each participant provided written consent to participate in this study, with consents stored in study files and approved by the University of South Australia: Ethics protocol 022806. In the light of results and feedback, all items were reassessed and 30 items were selected for final inclusion. These included three with a focus on the local South Australian ecological environment, ten relating to both the South Australian and Australian environments in general, with the remaining seventeen items relating to global ecological and natural system processes and functions.

Members of the SRG undertook the 30 item test and were scored using the pre-established answer values (-2 to 4). The group then reviewed each item once again, including for structure and relevance of questions, ordering of answer options, clarity of language and areas of potential confusion. In this way the ultimate version of each question was determined. It was acknowledged that the item pool represented a very small sample of possible items that could be constructed from the diverse content universe [39]. During this final review process some items were slightly modified and, in the light of these revisions, SRG scores were moderated and benchmarked to be indicative scores for Band One. This represented scientists and educators expected to have very high levels of ecological knowledge and understanding. While scores achieved by the SRG were not appropriate for statistical analysis, moderated scores generally exceeded 90%.

The question of what constitutes mastery in this assessment was informed by the work of the SRG. Complete mastery (100%) was not considered to be possible for two primary reasons. Firstly, the subject matter ranged through many ecologically-related disciplines and no individual was likely to have a complete understanding of all the topics examined. Most individuals have specialized knowledge in domains related to their own occupation or interest and rarely have both breadth and depth of knowledge in other domains [51]. In addition, research into such a diverse content universe is in a constant state of evolution, and diversity is a characteristic of ecological thinking [52]. Therefore, while the items were agreed and validated by a range of subject specialists, areas of debate will persist. In their discussion of the issue of mastery, Hopkins and colleagues explain: “When concepts are complex, we are usually in a continual state of growth in our understanding and never arrive at total mastery” ([39] p186). In summary, there is no perfect knowledge, nor indeed is there one kind of knowledge [53]. Consequently, 100% mastery was considered unrealistic and 90%, the average score achieved by the SRG during the moderation process, was considered to indicate an extremely high level of mastery of the knowledge and understanding tested in this assessment.

The final assessment [S1 Appendix] was in effect a “knowledge-based exam” ([41] p61) justifiable through the requirement for empirical analysis. It was developed through research, testing, reference to current assessment pedagogy and included an ongoing review process by a reference panel of senior scientists and educators. The instrument was a carefully structured, formal assessment tool applicable to western, science-based adult communities, with the capacity to generate information and illuminate patterns of ecological knowledge and understanding in the target community. We did not seek to capture either an individual’s or a community’s full knowledge or understanding of natural systems. We aimed instead to gather a broad range of data and make a general assessment of the state of knowledge and understanding within the self-selected sample adult community at the time. Embodied within this approach was a commitment to a thorough and rigorous process of research, development, testing and refinement.

### Benchmarking process

The instrument was then tested for its capability to differentiate between levels of knowledge and understanding among and between different groups with expected differences. Two distinct groups labelled as Band Two and Band Three (as distinct from the Band One SRG) were tested. Band Two consisted of 25 tertiary educated practitioners working in ecologically related fields while Band Three consisted of 32 local government manual workers without tertiary education. Participation was voluntary and with informed consent, covered by Ethics protocol 022806.

Band Two members were individually selected. They were known to the research team as professionals or practitioners working in a range of environmental fields such as education, management, consultancy and landscape design. Each had undertaken higher education in science, environment and conservation, natural resource management or a related field. Ages ranged from late twenties to mid-seventies, with all but one having lived in Australia for 20 years or more. The gender distribution was 13 males and 12 females. They were considered by the research team to be representative of individuals with expected high levels of ecological literacy although not necessarily as high as the scientific reference group.

Band Three comprised local government parks and gardens staff working in manual, outdoor occupations within a peri-urban jurisdiction. Typical work responsibilities and duties included pest control, arboriculture, sports field maintenance and park maintenance. Most members had completed secondary school with many having some subsequent vocational education or training in horticulture or related fields, although none had university level education. Ages ranged from early twenties to early sixties with all having lived in Australia for at least 20 years. The group included 29 males and three females. While their work entailed exposure to, and possible interest in, ecological processes and functions, they were considered to be representative of individuals with low to moderate levels of ecological knowledge.

For both groups, paper-based assessments were undertaken by individuals within supervised classroom-type situations. We expected that scores would vary between individuals and groups and that there would be a significant difference between the scores of Band Two and Band Three, with Band Two generally achieving higher scores. We also anticipated that there would be some overlap between the scores of the two bands.

To determine the expected scores to be achieved through random answer selections, 10,000 computer-generated test completions selected answers at random. For random answers the 10,000 iterations provided an expected score of 32/120 (120 being the maximum possible score and -60 being the minimum possible score) with a 95% confidence interval of 10-52/120.

### Application of the instrument

The assessment instrument, comprising 30 test questions and 40 socio-demographic, psychographic and lifestyle questions, was applied in the form of an online ‘survey’.

#### Socio-demographic and psychographic correlates

A range of socio-demographic and psychographic questions was designed to capture as much relevant and useful information as possible in order to maximize the opportunity to establish relationships between eco-literacy levels and the personal characteristics of respondents. These included a combination of scaled and multiple response questions (Table 1). These were constructed from a synthesis of questions used by the Australian Bureau of Statistics in collecting Census data [54] and similar data collection surveys. In addition, questions with specific relationship to the survey subject matter were developed. This section consisted of 40 items including 35 personal characteristic items and five open-ended, text based items. While the results of the social and demographic correlates are not the subject of this paper, the items were included in the online application of the instrument.

**Table 1:** Summary of the focus of socio-demographic and psychographic survey questions.

Socio-demographic	Lifestyle and psychographic
Gender	Time outdoors during childhood
Age group	Importance of nature in childhood household
Region of birth	Time outdoors during adulthood
Number of years in Australia	Importance of spending time outdoors to enjoyment of life
Number of years in South Australia	Importance of nature in current household
Aboriginal/Torres Strait origin	Time spent involved in garden/park/nature reserve
Australian citizen/permanent resident	Quantity of food consumed grown by household
Postcode	Quantity of food consumed produced within SA
Highest completed level of formal education	Frequency of participation in volunteer environmental activities
Subjects studied during senior secondary school	Most important contributors to knowledge/understanding of natural environment
Subjects studied as part of formal qualifications	Level of interest in improving knowledge/understanding of natural environment
Disciplines of completed formal qualifications	Extent of use of ecological knowledge in work
Current student status	Membership of associations/organisations related to environment/science
Current employment status	
Total annual income range	
Work role	
Field or industry of work	
Primary occupation	
Employment sector	
Place of childhood/growing up	

The Eco-literacy survey was available for six weeks during September and October of 2012, using the survey software Survey Monkey Platinum Version. It was self-selecting, self-administered, voluntary and anonymous. Because of the subject matter, the length of the survey and the level of intellectual demand contained within the questions, it was not intended to target a representative population sample. People without interest in the natural environment are unlikely to voluntarily undertake an ecological survey, especially one that requires considered thought and time investment.

The survey instrument was distributed through groups and organisations including a number of industry and professional associations, government agencies, non-government organisations and volunteer groups. Some of these bodies had a specific environmental affiliation while others were linked to fields such as education, science, planning, primary production, recreation and health. Each organisation was contacted with an introductory letter via email to ascertain willingness to distribute the survey. Leaders were invited to send the survey invitation and hyper-link to their employees, colleagues, members or associates. While most were willing to assist, some declined. As is often the case with web-based material, the survey invitation was further distributed by interested individuals. Research was approved by the University of South Australia: Ethics protocols: 022806 and 027205.

## Results

Results are described in two parts. Part one describes results from the benchmarking process, which led to the establishment of a range of standards of ecological knowledge and understanding. Part two describes overall results from the online application of the instrument.

### Part one: results of benchmarking process

Analysis of the data generated by Band Two and Band Three scores included a range of tests: the Anderson Darling normality test; an estimate of the level of internal consistency of the thirty items using Cronbach’s Alpha; an Independent Sample T-test to ascertain whether or not the means of the two bands were statistically different; and a randomisation test to determine the expected scores as a result of random answers.

**Fig 1:**
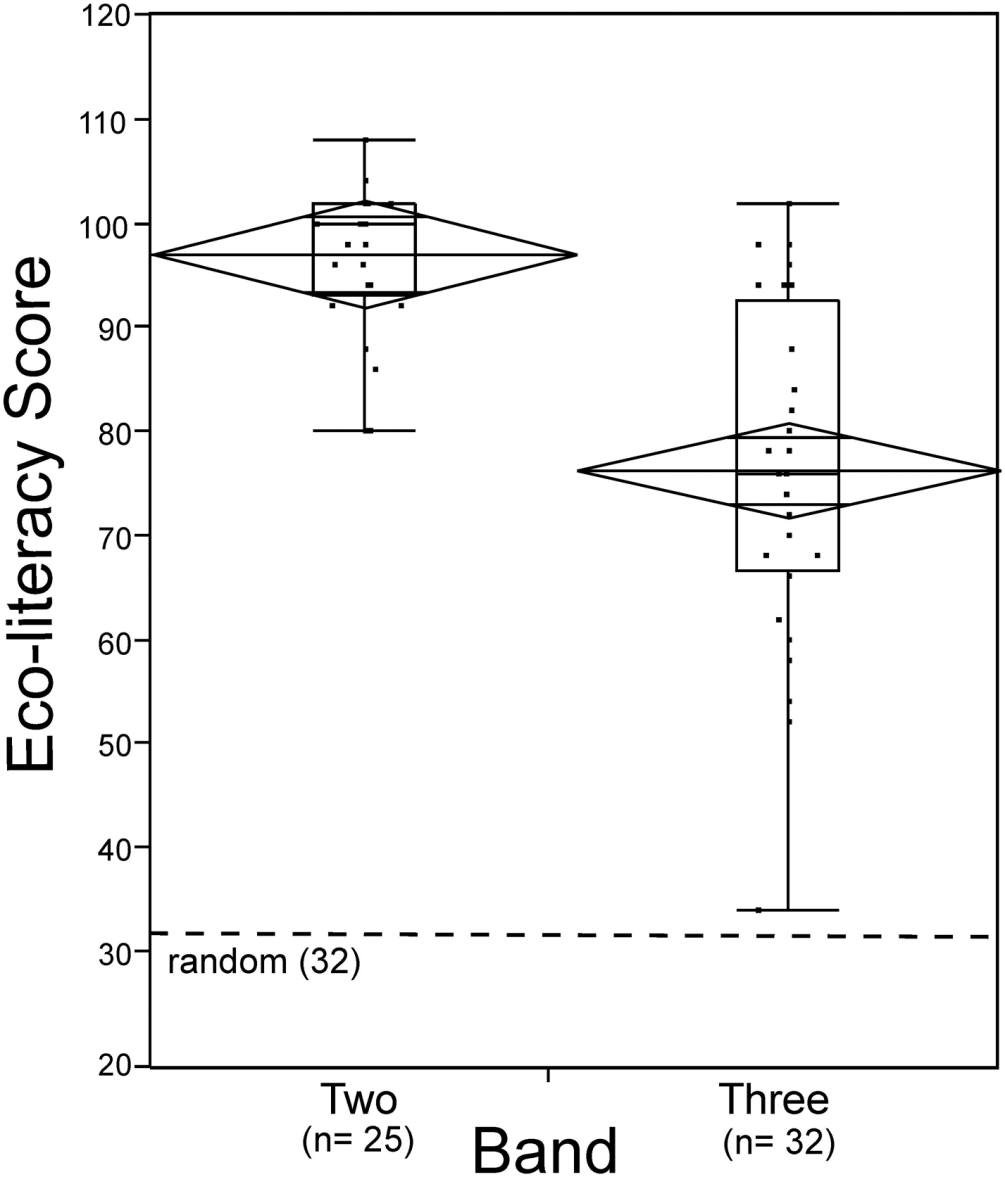
Box plots show scores for Band Two and Band Three respondents. Each box represents the middle 50% of the data set. The vertical lines show the range of the upper and lower quartiles. The median, or 50th percentile, is represented by the horizontal line within each box. The upper and lower edges of the box indicate the 75th and 25th percentile. The diamonds represent the 95% confidence interval. The dots represent actual score values of individual survey respondents.

Results showed a significant difference between the scores of Band Two and Band Three respondents (Fig 1). The total possible score for each respondent was 120: Band Two (mean = 97.12, standard deviation = 7.53, range = 28, sample size = 25); Band Three (mean = 76.31, standard deviation = 15.81, range = 68, sample size = 32). Band Two generally achieved higher scores although there was some overlap in scores between these two groups. Band Two scores were also more compact than those of Band Three.

Data for each group was normally distributed (*p* > .05). Cronbach’s alpha showed the internal consistency of the items to be acceptable (α = 0.78). Cronbach’s alpha is a coefficient of reliability (or consistency) and tends to increase as the intercorrelations among test items increase. It is generally considered that a Cronbach’s alpha value of 0.70 or higher indicates an acceptable degree of test score reliability [55–56]. An Independent Sample T-test showed a significant result (*t* = 6.55, *P* < .001, *df* = 55) and the point estimate of the difference between means was 20.81. The standard error of this difference was 3.18 and the confidence interval for the difference was 14.42–27.20. The effect size for the result was very large (1.68) with a 95% confidence interval of 1.07–2.29.

#### Aspirational scores and standards

**Fig 2:**
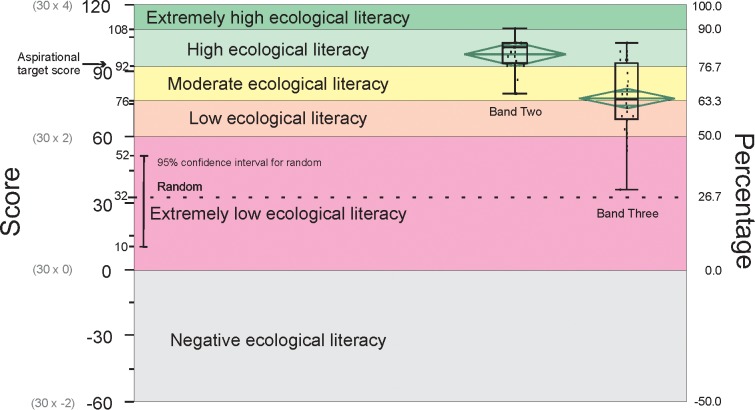
Standards system for ecological knowledge and understanding determined through the benchmarking process. Scores out of 120 are also shown as percentages. The expected score for random choice answers is 32. Band Two and Three results are shown in relation to the standards.

We considered how to determine reasonable and unreasonable standards of ecological knowledge and understanding for South Australian citizenry. The assessment tool was able to indicate degrees of mastery within and between three banded sets of scores, and we had an expected range of scores for completely random answers. It was possible to convert these into a logical scale that described a range of levels (Fig 2).

We identified an overlap of scores between Band Two and Band Three (Fig 1). The 25th percentile score for Band Two (93/120) and the 75th percentile score for Band Three (92.5/120) were within the same single answer range. Anyone achieving a score of 92/120 had answered a minimum of 16 questions correctly (16 x 4 = 53.33%) making up the difference with 14 questions partially correct (14 x 2). Achieving this score with other answer combinations required more than 16 questions to be answered correctly. This score (92/120), the minimum achieved by 75% of Band Two respondents and 25% of Band Three respondents, was therefore considered as our ‘aspirational target’ for a reasonable level of ecological comprehension.

The median score for Band Three was 76/120 (63.33%). Using this as a benchmark, we considered a ‘moderate’ standard to be indicated by scores of 76/120 and higher. The aspirational target of 92/120 (76.66%) also served as the starting point for a ‘high’ standard of ecological literacy. Two Band Two respondents scored 108/120 (90%) and most of the six SRG members scored equal to this or higher. While scores between 92/120 and 108/120 (76.66% and 90%) indicated a ‘high’ level of knowledge and understanding, scores of 108/120 (90%) and above were considered to be ‘extremely high’.

Our low range can be summarised as follows. All scores below 76/120 (63.33%) were considered ‘low’. A score of -60/120 (30x-2) equates to -50% and was the lowest score possible. This score was the ultimate indicator of negative ecological knowledge. Any score between -60/120 and 0/120 (-50% and 0%) indicated negative knowledge. Scores between 0/120 and 60/120 (0% and 50%) indicated ‘extremely low’ knowledge. It was not acceptable that a score within the 95% confidence interval for random answers (10-52/120) indicated even a ‘low’ level of knowledge. It was the case, however, that answering all 30 questions with partially correct answers would receive a score of 60/120 (50%), our starting point for consideration of a ‘low’ score. This was justified because any respondent scoring less than 60/120 had insufficient knowledge and understanding to balance incorrect answers with correct answers across the 30 questions. Scores between 60/120 and 76/120 (50% and 63.33%) were considered ‘low’.

Using this scoring system, 2/25 Band Two respondents rated as ‘extremely high’, 19/25 rated as ‘high’ and 4/25 rated in the ‘moderate’ range. Of Band Three respondents, 8/32 rated as ‘high’, 9/32 rated as ‘moderate’, 11/32 rated as ‘low’ and 4/32 rated as ‘extremely low’.

### Part two: results of online application of the instrument

The assessment and survey was commenced by 1,390 respondents and submitted with valid data by 1,010 respondents, a completion rate of 72.7%. The Survey Monkey software output the responses into an MS Excel spread sheet. Scores were calculated based on answers to the 30 multiple-choice questions [S1 Master Data]. Each respondent achieved a score out of 120 derived from the variable credits awarded for each answer. Theoretically scores could range from a minimum of negative 60 to a maximum of 120. Scores achieved through completely random answer sets, based on 10,000 iterations, ranged between 10 and 52 within a 95% confidence interval, with an expected score of 32.

**Fig 3:**
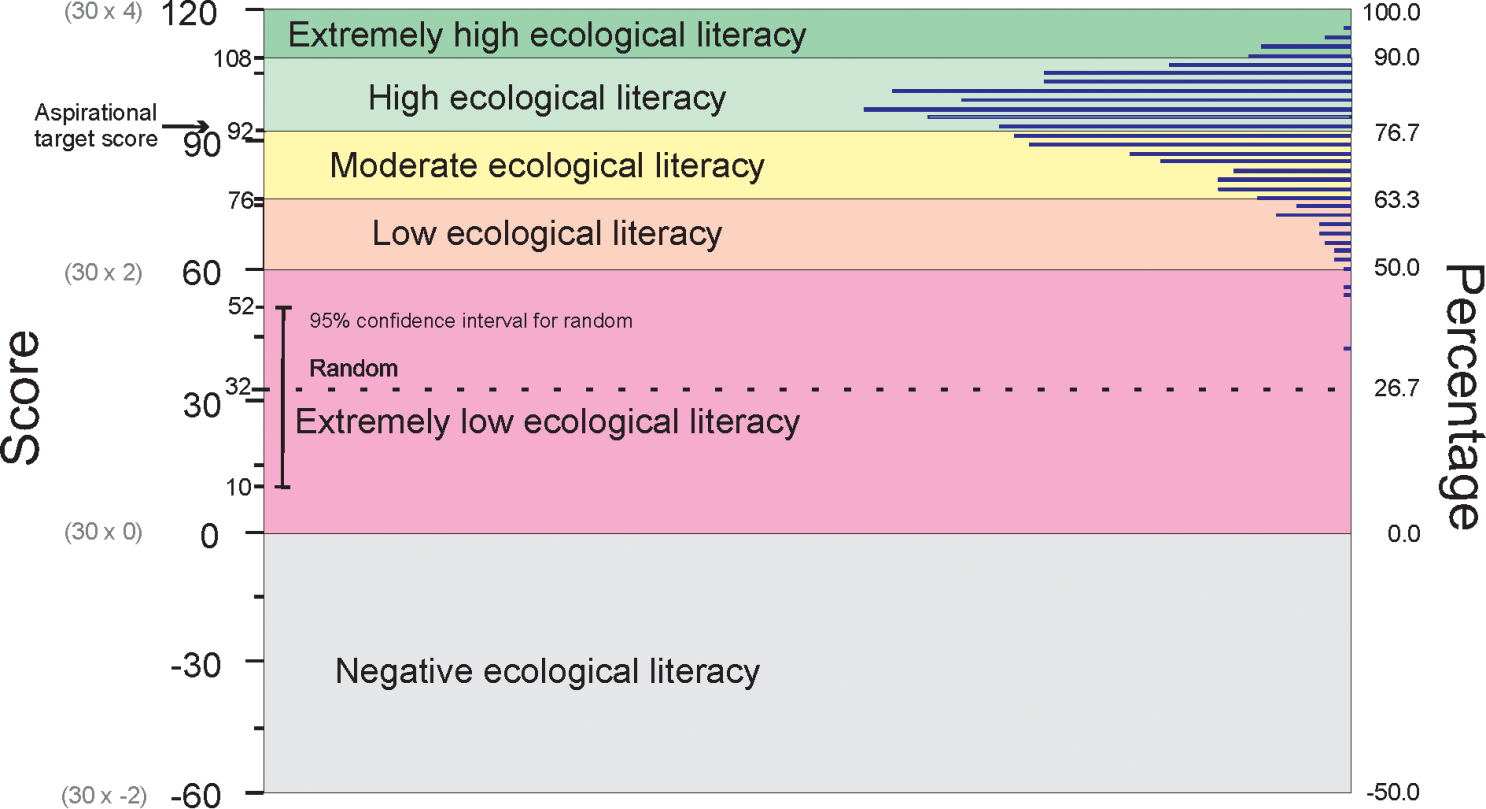
The total range of 1,010 valid data sets distributed within the eco-literacy standards system.

Data was collated and scores were distributed within ranges according to the eco-literacy standards system (Fig 3). Within the 1,010 individual data sets, scores ranged from 42/120 (35%) to 114/120 (95%). The average score of all assessments was 93/120 (93.02 ± 0.31) (77.5%). This was one point above the aspirational target score of 92/120 (76.7%). Of the 1,010 respondents, 45 (4.5%) achieved scores in the ‘extremely high’ range, 596 (59%) achieved scores in the ‘high’ range, 316 (31%) achieved scores in the ‘moderate’ range, 50 (5%) achieved scores in the ‘low’ range, and three (0.3%) achieved scores in the ‘extremely low’ range. The highest score achieved was 114/120 (95%) and the lowest score achieved was 42/120 (35%). The lowest score (42/120) was the only achieved score which fell within the 95% confidence interval for random answers (10-52/120).

## Discussion

Results from the benchmarking process indicate that ecological knowledge and understanding can be measured using this assessment instrument. Clear and significant differences between each Band, along with the wide range of scores, indicate unequal levels of mastery between both individuals and groups (Fig 1). The Band One scores of the SRG were highest (average 90%), an expected result as SRG members shared the highest levels of education in related disciplines, professional engagement in research and involvement in question development. Band Two respondents performed consistently better (average 81%), with less variation in scores, than Band Three (average 64%). A small number of Band Three participants, however, performed very well, with 25% scoring in the ‘high’ range. This suggests that while education and occupation are likely to have some correlation with the scores, they may not be the only related factors. This is an important result as it suggests that ecological literacy need not be the exclusive domain of the highly educated or professionally employed. It also demonstrates that the assessment instrument is able to capture mastery of ecological knowledge and understanding that has not been achieved through tertiary education or professional employment streams.

The online application of the instrument to a wider audience delivered scores that were generally moderate to high. Only 5.3% of respondents achieved scores in the low or extremely low ranges. This is considered to reflect two related factors in particular: the self-selecting nature of the survey and the high salience of the topic to the respondent group. While respondents did not form a representative sample of the South Australian adult population, the highest margin of the sample group was likely to reflect the most ecologically literate within the population at large. Many of the respondents in the survey were well-educated in environmental sciences, were engaged in ecologically-related paid or volunteer work or had environmental interests. These factors contributed to their participation in the survey itself. The highest scores within the total sample (45 of 1,010) were equal to or greater than the Band One scores of the Scientific Reference Group. Therefore, it is considered that the collective ecological knowledge and understanding within the total sample is unlikely to be significantly greater within any other groups or population samples within the South Australian community. On the other hand, respondents who scored within the lowest margin of the sample are still likely to be more ecologically literate than other population groups within the community because they represent the ‘low’ end of a self-selecting sample of people sufficiently engaged and interested to undertake the assessment and questionnaire.

The average score achieved in the test was 93/120, with 63.5% of respondents realizing the aspirational target of 92/120. This indicates that the target is indeed attainable. It is encouraging that a majority of this sample population demonstrated knowledge and understanding equal to or above an acceptable level.

### Test development

The assessment instrument is the product of a rigorous and collaborative process of research, testing and refinement. It is a multiple-choice test that can measure partial knowledge as well as the absence of knowledge with defensible results. A sensitive system of partial credits recognizes answers that demonstrate varying levels of mastery of the subject matter, and captures a higher resolution of knowledge and understanding than is possible in a standard test. While the test reflects a broad canvassing of ecological concepts, it does not require specific formal knowledge of these concepts, but rather an effective understanding of their implications. It is able to capture knowledge and understanding acquired in diverse ways while respecting and valuing formal education. Furthermore, the test can deliver a determinant of ‘extremely low’ through to ‘extremely high’ standards, including an identified aspirational target score. These standards can be used to benchmark individuals, groups, organizations and communities within the South Australian population.

The instrument satisfies the perceived need for both local place-based knowledge and an understanding of global systems, including an understanding of the interface with human society. The combination of items relating to local, national and global environments and systems means that results are likely to indicate an across-the-board comprehension and awareness. While acknowledging the diversity in scientific thinking, including within the field of ecology, we consider the comprehensive blend of place-related material, ecological principles and topical representations to be a feature of the instrument, providing a potential model for similar assessments in other communities.

### Development of a standards system

The test development process facilitated the establishment of a standards system that enabled an ‘a priori’ aspirational target to be determined as well as criteria for levels of achievement. This standards system provides a well-defined and consistent method for identifying an indicative level of ecological knowledge and understanding in individuals who undertake the assessment. The distribution of scores into a standards system enables efficient processing of data while maintaining the integrity of the process. This system facilitates comparisons between respondent characteristics and various levels of achievement, therefore allowing closer investigation of sub-groups within a larger population.

In addition, the establishment of an aspirational target score provides a simple criterion by which to measure the indicative ecological knowledge and understanding of an individual or group. It also provides a benchmark from which to investigate the specific characteristics of those who did and did not achieve the aspirational target. Of the total sample of 1,010 respondents who submitted valid responses in this survey, 63.5% achieved the aspirational target score. This is an encouraging result and reveals that, of the individuals from the self-selecting respondent group who undertook the assessment, the majority demonstrated high ecological literacy.

### Strengths and limitations

Due to the parameters and nature of the study, the instrument has both specific strengths and limitations. It has several major strengths: the advisory scientific reference group included a panel of senior and respected scientists and environmental educators with diverse expertise who applied rigour to question and answer construction; while the test reflects a broad canvasing of ecological principles, it does not require specific knowledge of these principles but rather an effective understanding of their implications; the combination of a local place-based approach with a regional and global systems approach allows capture of a broad comprehension of ecological systems; and it is a sensitivity test calculated using partial credits so that varying levels of comprehension can be accommodated and credited. Efforts have been made to achieve both a consensus of opinion while engaging a cosmopolitan approach to knowledge that “recognises its place- and context-shaped characteristics” ([53] p 563).

The instrument also has limitations. It is a feature of such a process that measured items are based on the values and perspectives of those involved in developing and designing the instrument. In addition, the field of ecology is constantly evolving and encompasses diversity in thinking which exposes test items to difference of opinion. The focus on western science may also be considered a limitation in that traditional ecological knowledge was not specifically incorporated. It is also acknowledged that the assessment instrument does not address all possible components of ecological literacy, such as attitudes and behaviours; it is concerned with the underpinning ‘knowledge and understanding’ component. It can be argued that attitudes and behaviours are as important, if not more so, as knowledge and understanding. Limited research has been conducted within Australia in the fields of attitudes and behaviours in any type of ecological context, and the establishment of a link with ecological knowledge and understanding has not been attempted. This is an area in which future research may contribute valuable insights.

A further limitation of the assessment was the potential for collusion among respondents. In this regard it was considered important to limit the period of availability while providing a reasonable window of opportunity for interested parties to gain access through the various distribution networks. Because of the self-selecting nature of the assessment it was considered reasonably unlikely that respondents would collude as the subject matter was likely to be of personal or professional interest and no penalty was associated with incorrect responses. The time period of six weeks was therefore considered suitable.

This survey was a non-probability survey. Non-probability surveys include self-selecting participants and do not have equal probabilities of selecting members within target populations. Therefore, it is acknowledged that results cannot readily be generalised to the wider population. The population sample was dominated by the most interested and engaged sections of the community and, despite the wide range of scores achieved, on average the respondents performed well, demonstrating that interested and engaged people are likely to have a reasonable level of ecological knowledge and understanding. We fully expect that individuals for whom the subject matter has less appeal would also have less connection with natural systems and would not achieve such high scores.

Web-based delivery, while contributing to efficiency, can restrict accessibility. Although increasing numbers of people use computers and have access to the internet, not everyone has the skills, experience or sufficiently reliable access to undertake web-based surveys. Online survey respondents are unlikely to be a random part of the population [57]. Some groups within the community are therefore more likely than others to participate in web surveys and this can result in biased samples [58].

Regardless of its limitations, the assessment structure is founded on the scholarship and experience of many. It has achieved its goal of being able to capture knowledge and understanding acquired in diverse ways while respecting and valuing the importance of science and formal education. Development has involved a collaborative process based on review and feedback, with a focus on local and global ecological systems and how they function and support life, including humanity. Close attention has been paid to the work of foremost thinkers and practitioners whose concerns include the essential requirement of contemporary human communities to “understand the basic principles of ecology, and to live accordingly” ([26] p 2).

### Wider applicability

This assessment is pertinent to a particular spatial and temporal context. The nature of ecological literacy is that it encompasses and reflects what is known and understood about ecological systems in specific places and at specific times. There is opportunity, however, for the test to be modified and validated for application with other target groups in alternative communities in the future. In its current form the test is valid for and applicable to individuals and groups within South Australia. However, the method by which the test was developed can be replicated and a similar process could be employed to develop a valid assessment for other populations. While acknowledging the theoretical western scientific framework of the assessment process, it was our intention that the instrument focus on knowledge able to be acquired in diverse ways and from multiple sources, including other than formal educational settings. However, it may be possible and appropriate to integrate traditional ecological knowledge into such an assessment process.

## Conclusions

Evaluation of the level of community understanding of ecological systems may have a key role to play in the process of determining how current and future populations can live more sustainably on Earth. We have shown that it is possible to assess and describe indicative levels of ecological knowledge and understanding within an adult community. Through research, consultation, testing and analysis we have developed a valid and reliable assessment instrument with demonstrated capacity to discriminate between differing levels of ecological comprehension. Furthermore, the test can deliver a determinant of ‘extremely low’ through to ‘extremely high’ standards, including an identified aspirational target score. These standards can be used to benchmark individuals, groups, organizations and communities within the wider population.

Application of the instrument yielded considerable data for analysis. The ecological knowledge and understanding of a self-selecting sample of 1,010 adults within the South Australian community was shown to be generally moderate to high, with a small percentage in the low ranges and a small percentage in the extremely high range. Examination of the relationships between scores and socio-demographic and psychographic characteristics has generated detailed analyses of the data, presented and discussed in further papers. This work contributes to a deepening understanding of factors associated with ecological knowledge and understanding within an industrialised and urbanised Western society.

## Supporting Information

S1 Appendix (DOCX)

S1 Master Data (XLS)
