# Supplementary material for: Quantifying Ecological Literacy in an Adult Western Community: The Development and Application of a New Assessment Tool and Community Standard
Source: PLoS One. 2016 Mar 3;11(3):e0150648. doi: 10.1371/journal.pone.0150648 (PMC4777481; doi:10.1371/journal.pone.0150648)
Supplement: S1 Appendix — (DOCX) [file pone.0150648.s001.docx]

**S1 Appendix. Eco Literacy Assessment Questions.**

Value of credits and partial credits included

Q1. The Leafy Seadragon is South Australia’s marine emblem. These fish can often avoid predators because they

• are aggressive. (-2)

• look like seaweed. (4)

• shelter in caves and crevices. (0)

• can swim rapidly. (0)

• can anchor themselves to vegetation. (2)

Q2. In South Australia trees contribute to the health of hydrological (water) systems because they

• shade the soil. (0)

• attract rain. (0)

• stabilise waterways. (2)

• provide essential calcium to waterways. (-2)

• keep the water table from rising to the surface. (4)

Q3. Many marine species inhabit seagrass meadows for some or all of their lives and more fish live among seagrasses than over adjacent mudflats. This is mainly because

• dead and decaying seagrasses are the basis of the food web for many fish. (4)

• small fish are better able to escape from predators if they can hide in seagrass. (2)

• seagrass meadows are fish nurseries. (2)

• most fish living in seagrass eat seagrass. (0)

• plankton eaten by fish only live in seagrass meadows. (-2)

Q4. What is the major contributing factor to the endangerment of native animal species in the Mount Lofty Ranges?

• Roadways (0)

• Competition with pests (2)

• Use of pesticides (2)

• Loss of habitat (4)

• Harvesting for pet food (-2)

Q5. The most important single action we can take to encourage small native birds back into parks and gardens is to

• protect them from dogs. (2)

• plant low and medium-sized, dense, bushy shrubs. (4)

• plant tall gum trees and native grasses. (0)

• provide fresh water. (2)

• clear away understorey and groundcover vegetation. (-2)

Q6. The removal of most of Adelaide’s coastal dunes is now considered to have been a mistake because sand dunes

• protect inland areas from wind. (2)

• are high in nitrogen. (-2)

• provide habitat for many animals and birds. (2)

• supply and replenish sandy beaches. (4)

• help protect seagrass meadows. (0)

Q7. Prevention of flooding has negatively affected many River red gum trees because

• River red gums typically germinate after a flood. (4)

• River red gums need to be flooded most of the time. (-2)

• flooding flushes and renews the soil, bringing fresh nutrients. (2)

• River red gums need to be flooded every few years. (0)

• flooding attracts the wildlife the River red gums rely on. (0)

Q8. Fire can be important for the regeneration of native plants in Australia. This is mainly because

• fire eliminates the diseases that affect native plants. (0)

• heat and smoke trigger seed germination in some plants. (4)

• fire kills the carnivores that eat the plants. (-2)

• fire reduces undergrowth and allows light to reach the soil. (2)

• ash and charcoal change the soil chemistry to promote germination. (2)

Q9. Seaweed and seagrass washed up on beaches are important because they

• help prevent foreshore erosion. (2)

• help prevent high tides from reaching nesting shorebirds. (0)

• absorb methane and so help regulate greenhouse gases. (-2)

• provide food for many animals. (4)

• provide habitat for shorebirds. (2)

Q10. Many of the plants introduced to southern Australia during the past 200 years have become environmental weeds, resulting in

• changes to fire intensity and frequency. (2)

• suitable habitat for native fauna. (0)

• reduced levels of nitrogen available in soils. (0)

• decline in the diversity and abundance of native plants. (4)

• less evaporation from soil. (-2)

Q11. The best habitat for sheltering hollow-dependent wildlife is found in

• dead trees. (2)

• saplings. (-2)

• live, hollow-bearing eucalypt trees. (4)

• live, hollow-bearing exotic trees. (2)

• roofs, sheds and bridges. (0)

Q12. Dingoes are having a significant impact on the environment because they

• compete with native animals for water. (0)

• consume native plants. (-2)

• threaten the survival of native marsupials. (2)

• help control populations of cats and foxes. (4)

• move south as northern areas become hotter and dryer. (0)

Q13. The Earth’s atmosphere is critically important for life because it

• contains the moisture that becomes rain. (2)

• traps heat, provides oxygen and protects from radiation. (4)

• allows light to reach the surface of the Earth. (0)

• allows greenhouse gases produced on Earth to be released into space. (-2)

• regulates the air pressure systems that influence weather. (2)

Q14. The global climate is most influenced by

• micro-climates. (0)

• the Sun. (4)

• greenhouse gases. (2)

• the temperature of the major oceans. (2)

• lightning. (-2)

Q15. The extent and intensity of rainfall is most affected by

• prevailing winds. (2)

• altitude and landform. (2)

• ocean surface temperature and currents. (4)

• galactic winds. (-2)

• availability of fresh water for evaporation. (0)

Q16. What is the most effective way to conserve life on Earth?

• Collect DNA from each species and freeze it for the future (0)

• Eliminate the organisms that spread disease. (0)

• Preserve all species in zoos and gardens. (-2)

• Establish many large conservation parks. (2)

• Conserve a representative proportion of ecosystems and habitats. (4)

Q17. Micro-organisms are invisible to the naked human eye. Micro-organisms

• are dangerous as they infect plants and animals with disease. (2)

• have minimal benefits for life on Earth. (-2)

• are found mostly in water. (0)

• always have short life spans. (0)

• are vital for decomposition of dead matter. (4)

Q18. Human medicines originate mainly from

• fungi and algae. (2)

• peat bogs. (-2)

• micro-organisms. (2)

• plants. (4)

• chemical compounds manufactured in laboratories. (0)

Q19. Ecosystems perform a variety of beneficial functions (sometimes called ecosystem services) through the interactions of organisms with their environments. Essential services provided by ecosystems include

• providing healthy environments for recreation, relaxation and fitness. (2)

• controlling diseases. (2)

• breaking down wastes and recycling nutrients. (4)

• providing an inexhaustible supply of materials. (-2)

• clearing away dust and smoke particles. (0)

Q20. Coastal marshes and mangrove forests provide important ecosystem services because they

• produce food and materials for human use. (2)

• recharge ground water. (2)

• absorb high levels of greenhouse gases. (0)

• buffer the land against ocean storm surges and prevent coastal erosion. (4)

• are suitable for waste dumps and can be used to form artificial land. (-2)

Q21. A healthy garden can be described as an ecosystem because

• it is self-contained and separate from the outside world. (0)

• it supports a web of biological relationships. (4)

• the cycling of water and nutrients, as well as the plant-animal interactions, are essential to the garden’s survival. (2)

• it contains healthy plants and water. (0)

• it supports the economy by using resources. (-2)

Q22. Deserts are arid ecosystems that have little surface water most of the time. In deserts

• most species are concentrated in run-off areas. (2)

• species react quickly to sudden changes in water and food abundance. (4)

• dead plant and animal matter is mainly processed by earthworms. (-2)

• plants and animals are short-lived with a high population turnover. (0)

• the number of species increases after rain. (0)

Q23. Some ecosystems are more sensitive than others to a changing climate. In a warming and drying climate, the most sensitive ecosystems are

• woodlands. (2)

• rainforests. (4)

• savannahs. (0)

• wetlands. (2)

• deep ocean beds. (-2)

Q24. Carbon is most effectively absorbed by

• wooden building materials and furniture. (-2)

• marine plants and oceans. (4)

• crops and pastures. (0)

• soil. (2)

• forests. (2)

Q25. An urban heat island is a form of micro-climate that can develop in towns and cities. An urban heat island

• forms mainly on busy roundabouts and median strips in transport corridors. (-2)

• forms when dark, solid surfaces absorb solar radiation which is then released at night. (4)

• is only a problem in hot weather because the land and air cool in winter. (0)

• forms when heat released from factories, air conditioners and vehicles is trapped between buildings. (2)

• keeps towns and cities warmer in winter. (0)

Q26. Extinctions, the loss of species altogether, are a natural part of evolution of life on Earth. Current extinctions

• involve animals and plants unable to relocate when their habitat is lost. (2)

• will help reduce overcrowding on Earth. (-2)

• mainly involve species unable to adapt quickly to urban environments. (2)

• are spread fairly evenly around the Earth. (0)

• are occurring mainly in areas rich in species and where habitat destruction is high. (4)

Q27. Currently the greatest threat to tropical coral reefs is

• suffocation and starvation of sea life by plastic waste. (0)

• acidification of seawater affecting formation of the coral skeleton. (2)

• warming of surface water temperatures which leads to coral bleaching. (4)

• overfishing and fishing practices that use cyanide and dynamite. (2)

• cooling of surface water temperatures due to melting ice. (-2)

Q28. The melting of sea ice in the Arctic is decreasing seal populations. This is mainly because

• polar bears find it easier to capture seals in larger areas of open water. (-2)

• the roofs of seal snow dens collapse more frequently and pups are crushed. (2)

• warmer waters bring food species that seals are not adapted to feed on. (0)

• warmer waters bring in new predators that feed on seals. (0)

• as ice melts more quickly pups are exposed to predators on land and in water. (4)

Q29. Night light radiating from cities and towns is sometimes called light pollution. Light pollution can have significant effects on nature as it

• leads to larger populations of bats. (0)

• interferes with the natural behaviours of nocturnal animals. (4)

• prevents micro-organisms from eating surface algae, leading to algal blooms. (2)

• means that micro-organisms can spread disease more rapidly. (-2)

• reduces the visibility of stars. (2)

Q30. The five pictures were taken in South Australia. Which shows the most sustainable ecosystem?

(pictures not included here)
